# Modulation of SNARE-dependent exocytosis in astrocytes improves neuropathology in Huntington's disease

**DOI:** 10.1242/dmm.052002

**Published:** 2024-11-11

**Authors:** Annesha C. King, Emily Payne, Emily Stephens, Jahmel A. Fowler, Tara E. Wood, Efrain Rodriguez, Michelle Gray

**Affiliations:** ^1^ Graduate Biomedical Sciences Neuroscience Theme, University of Alabama at Birmingham, Birmingham, AL 35294, USA; ^2^ Department of Neurology, Center for Neurodegeneration and Experimental Therapeutics, University of Alabama at Birmingham, Birmingham, AL 35294, USA; ^3^ Graduate Biomedical Sciences Biochemistry and Structural Biology Theme, University of Alabama at Birmingham, Birmingham, AL 35294, USA

**Keywords:** Huntington's disease, Astrocytes, BACHD, dnSNARE, SNARE-dependent exocytosis

## Abstract

Huntington's disease (HD) is a fatal, progressive neurodegenerative disorder. Prior studies revealed an increase in extracellular glutamate levels after evoking astrocytic SNARE-dependent exocytosis from cultured primary astrocytes from mutant huntingtin (mHTT)-expressing BACHD mice compared to control astrocytes, suggesting alterations in astrocytic SNARE-dependent exocytosis in HD. We used BACHD and dominant-negative (dn)SNARE mice to decrease SNARE-dependent exocytosis from astrocytes to determine whether reducing SNARE-dependent exocytosis from astrocytes could rescue neuropathological changes *in vivo*. We observed significant protection against striatal atrophy and no significant rescue of cortical atrophy in BACHD/dnSNARE mice compared to BACHD mice. Amino acid transporters are important for modulating the levels of extracellular neurotransmitters. BACHD mice had no change in GLT1 expression, decreased striatal GAT1 expression and increased levels of GAT3. There was no change in GAT1 after reducing astrocytic SNARE-dependent exocytosis, and increased GAT3 expression in BACHD mice was normalized in BACHD/dnSNARE mice. Thus, modulation of astrocytic SNARE-dependent exocytosis in BACHD mice is protective against striatal atrophy and modulates GABA transporter expression.

## Teaser

**Summary:** This study provides insight into the contribution of SNARE-dependent exocytosis from mutant HTT-expressing astrocytes to neuropathological changes in a mouse model of Huntington's disease.

## INTRODUCTION

Huntington's disease (HD) is a fatal neurodegenerative disease caused by a CAG expansion in the huntingtin (*HTT*) gene leading to a polyglutamine (polyQ) expansion in the widely expressed huntingtin protein (HTT) (MacDonald et al., 1993). Neuropathologically, HD is characterized by degeneration of striatal medium spiny neurons (MSNs), cortical pyramidal neurons and atrophy of other brain regions as disease progresses (McColgan and Tabrizi, 2018). Clinically, HD patients exhibit motor (chorea, dystonia), psychiatric (depression, irritability) and cognitive (impaired emotion recognition and executive functions) symptoms, which worsen progressively (McColgan and Tabrizi, 2018; Roos, 2010).

Astrocytes are specialized cells within the nervous system and perform numerous processes that are essential for proper nervous system function, including the regulation of potassium levels, synthesis and removal of neurotransmitters, and release of gliotransmitters (Anderson and Swanson, 2000). Astrocytes release amino acids, nucleotides, peptides and brain-derived neurotrophic factor to modulate neuronal function by intracellular Ca^2+^-dependent signaling (Parpura and Zorec, 2010; Reick et al., 2016). One of the hypothesized mechanisms by which astrocytes communicate with neurons is through Ca^2+^-dependent exocytosis using the soluble N-ethylmaleimide-sensitive fusion protein attachment protein receptor (SNARE) complex (Montana et al., 2004; 2006; Parpura and Zorec, 2010).

There is increasing evidence of astrocyte involvement in the pathogenesis of HD (Shin et al., 2005; Khakh et al., 2017). Astrocytes expressing mutant huntingtin (mHTT) increase neuronal cell death of wild-type neurons, whereas wild-type astrocytes protect mHTT-expressing neurons from death in cell culture (Bradford et al., 2009; Bradford et al., 2010). Astrocyte-specific expression of a N-terminal mHTT (a fragment driven by the GFAP promoter) with 160 polyglutamine repeats in mice cause an HD-like neurological phenotype (Bradford et al., 2009, 2010) and HD-like neuropathology (Shin et al., 2005).

A human bacterial artificial chromosome (BAC) containing the entire 170 kb human *HTT* locus flanked on the 5′ side by 20 kb of genomic sequence and on the 3′ side by 50 kb of genomic sequence was used to generate the BACHD transgenic mouse model. The BAC was modified by replacing exon 1 with a modified exon 1 containing 97 mixed CAA-CAG repeats flanked by LoxP sites (Gray et al., 2008; Menalled et al., 2009). The BACHD conditional transgenic mouse model exhibits motor, cognitive and psychiatric-like deficits that progressively worsen as the animal ages. Importantly, improvements in behavioral and neuropathological features in the BACHD mice were observed when mHTT was reduced in astrocytes (Wood et al., 2019). In addition, increased levels of extracellular glutamate were observed in cultures of cortical astrocytes from BACHD compared to wild-type astrocytes upon mechanical stimulation to evoke SNARE-dependent exocytosis (Lee et al., 2013). Such an increase in extracellular glutamate levels could lead to excitotoxicity and contribute to abnormalities in HD (Sánchez et al., 2008; Estrada-Sánchez and Rebec, 2012).

Altered striatal GABAergic and glutamatergic transmission coincident with the presence of behavioral abnormalities has been observed in mHTT-expressing mouse models (Smith-Dijak et al., 2019; André et al., 2010; Cepeda et al., 2007). The activity of GABAergic MSNs is regulated by extrastriatal glutamatergic and dopaminergic neurons and intrastriatal cholinergic and GABAergic interneurons (Kreitzer and Malenka, 2008). These inputs must be balanced for proper MSN function and striatal output. Astrocytic contribution to proper neurotransmission includes the synthesis, release and reuptake of amino acids, including glutamate and GABA, through transporters. There is a reduction in the level of *SLC1A2* mRNA, which encodes the astrocyte-enriched glutamate transporter GLT1/EAAT2 in postmortem HD patient striatal tissues (Arzberger et al., 1997), implicating astrocytes in excitotoxicity in HD. Furthermore, some mHTT-expressing mouse models showed a decrease in GLT1 expression, altered glutamate uptake and dysfunctional striatal neurons. The expression of mHTT selectively in astrocytes causes motor abnormalities (Bradford et al., 2009; 2010; Faideau et al., 2010; Ben Haim et al., 2015).

The levels of extracellular GABA are modulated by the GABA transporters (GATs) GAT3 and GAT1. GAT3 (encoded by *Slc6a11*) is expressed by astrocytes (Minelli et al., 1996), whereas neurons and astrocytes express GAT1 (encoded by *Slc6a1*) (Minelli et al., 1995; Jin et al., 2011). Alterations in GAT3 function have been implicated in grooming behaviors in wild-type mice when cytosolic Ca^2+^ was extruded from striatal astrocytes by a plasma membrane Ca^2+^ pump. Furthermore, in the R6/2 mHTT-expressing mouse model, blocking GAT3 function rescued abnormal grooming behavior exhibited by those mice (Yu et al., 2018). Taken together, these data implicate astrocyte-enriched amino acid transporters in the phenotypes observed in HD.

Previously, hGFAP.tTA/tetO.SNARE (dnSNARE) mice were used to determine whether reducing SNARE-dependent exocytosis from astrocytes would rescue the progressive abnormal behavioral phenotypes observed in BACHD mice (King et al., 2020). The dnSNARE mice contains tet operon (tetO)-driven cassettes that encode β-galactosidase (LacZ), enhanced green fluorescent protein (eGFP), and the cytoplasmic domain of vesicular protein synaptobrevin2/VAMP2 (dnSNARE) (Pascual et al., 2005). The expression of these transgenes (LacZ, eGFP and dnSNARE) is controlled by the presence/absence of the tetracycline transactivator, which is driven by the human glial fibrillary acidic protein (*GFAP*) promoter (hGFAP.tTA) in these mice. When there is no expression of the dnSNARE transgene, there is normal SNARE-dependent exocytosis. Differential effects on the behavioral phenotypes in the BACHD mice were observed, with an improvement in motor coordination and worsening of anxiety-like phenotypes after reducing SNARE-dependent exocytosis (King et al., 2020). These data clearly indicate a complex role for SNARE-dependent exocytosis from astrocytes in various behaviors. In the present study, we determined whether reducing astrocytic SNARE-dependent exocytosis would rescue abnormal neuropathological phenotypes in BACHD mice or alter the expression of proteins critically involved in maintaining proper neurotransmission. We performed neuropathological analyses and assessed astrocyte-enriched protein levels in BACHD mice after the reduction of SNARE-dependent exocytosis.

## RESULTS

### Neuropathological changes in BACHD/dnSNARE mice

There is selective degeneration of the striatal medium spiny neurons and, to a lesser extent, cortical pyramidal neurons, along with widespread atrophy and reduced brain weight in HD (Vonsattel et al., 1985; Halliday et al., 1998; Paulsen et al., 2006; Walker, 2007; Ross and Tabrizi, 2011; van den Bogaard et al., 2011). In some mouse models of HD, there is reduced brain weight and neuronal atrophy/loss (Menalled and Chesselet, 2002; Ferrante, 2009; Crook and Housman, 2011; Figiel et al., 2012). In BACHD mice, there is a significant reduction in brain weight and cortical and striatal volume (Gray et al., 2008; Wang et al., 2014; Wood et al., 2019). A previous study using BACHD mice crossed to dnSNARE mice revealed that a vast majority of cells co-labeled for GFP (which indicates proper activation of the transgenes) and S100β to label astrocytes in the cortex and striatum (King et al., 2020), indicating high selectivity for astrocytes in this model (Fellin et al., 2009; Grubišić and Parpura, 2017; Halassa et al., 2009; Pascual et al., 2005; King et al., 2020).

**Fig. 1.**
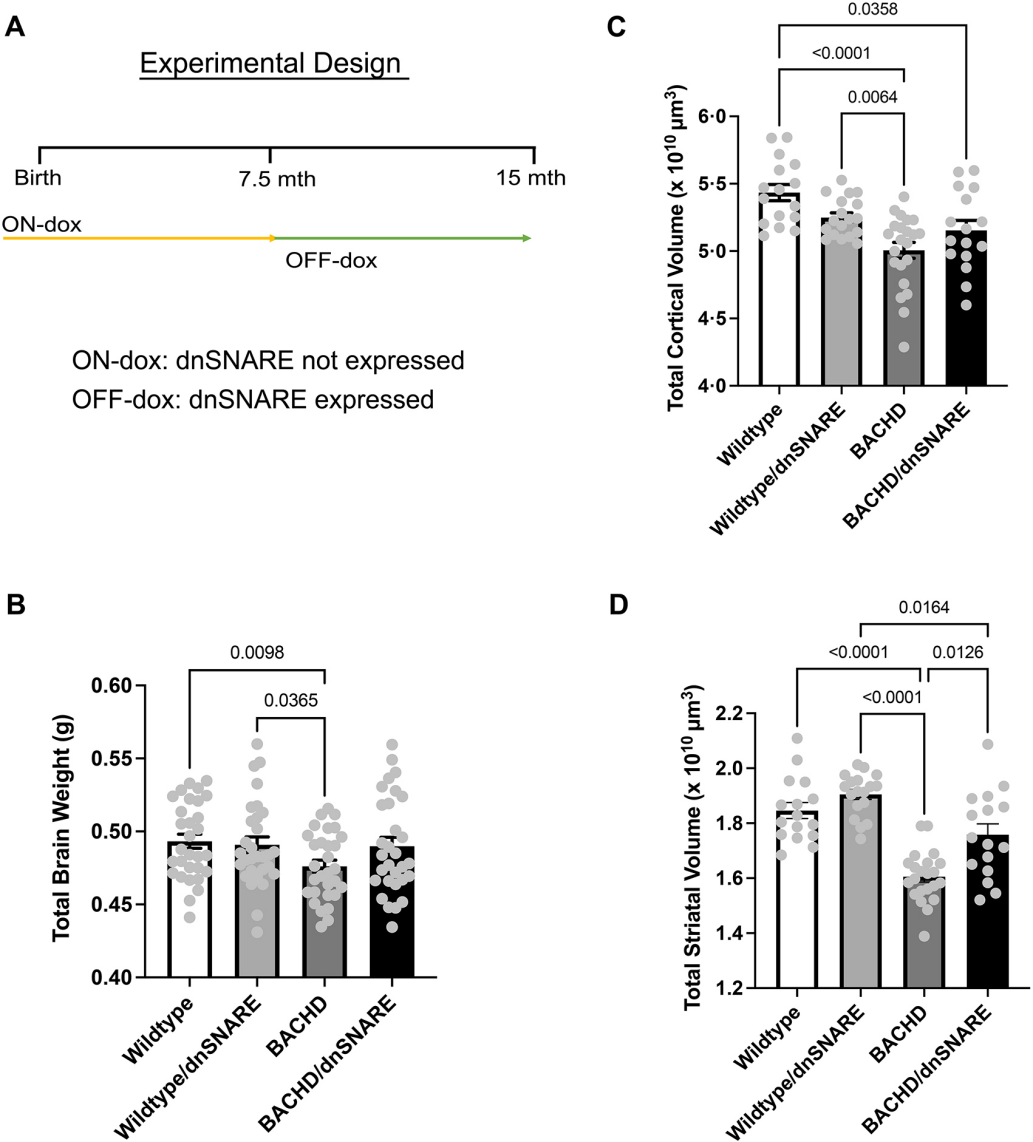
**BACHD/dnSNARE mice have increased striatal volume.** (A) Design of the doxycycline (dox) paradigm, showing the control of inducible astrocyte-specific dnSNARE expression. Mice were fed dox-containing chow for 7.5 months and removed from dox-containing food to allow transgene expression. (B) Total brain weight was assessed at 12-15 months of age (*n*=30/genotype). (C,D) Cortical (C) and striatal (D) volume was assessed at 12-15 months (*n*=16-22/genotype). Data are mean±s.e.m. Differences among the groups were assessed using Welch's ANOVA test followed by Dunnett T3 multiple comparison procedure. Non-significant *P*-values are not displayed on graphs. Refer to Table 1 for all *P*-values.

In order to assess neuropathological changes due to the reduction in astrocyte SNARE-dependent exocytosis, we analyzed total brain weight and volume of the cortex and striatum in BACHD/dnSNARE, BACHD, wild-type and wild-type/dnSNARE mice at 13-15 months of age, an age at which robust behavioral and neuropathological changes are observed (Gray et al., 2008) (Fig. 1A). It has previously been shown that dnSNARE expression in the cortex and striatum is found most prominently in astrocytes (King et al., 2020). Our analysis revealed a significant difference in brain weight between the genotypes [*F* (3,63.93)=2.911, *P*=0.0411] (Fig. 1B). BACHD mice had reduced brain weight compared to that of wild-type (*P*=0.0098) and wild-type/dnSNARE (*P*=0.0365) mice. There were also significant differences between the genotypes [*F* (3,35.02)=8.81, *P*=0.0002] for cortical volume (Fig. 1C). BACHD and BACHD/dnSNARE mice had reduced cortical volume compared to that of wild-type mice (*P*<0.0001, *P*=0.0358, respectively) (Fig. 1C). BACHD mice had reduced cortical volume compared to that of wild-type/dnSNARE mice (*P*=0.0064) (Fig. 1C), but the cortical volume of wild-type/dnSNARE mice was not significantly different from that of BACHD/dnSNARE mice (*P*=0.7987) (Fig. 1C). Thus, reducing SNARE-dependent exocytosis in BACHD mice does not cause statistically significant changes in brain weight or cortical volume.

**Table 1.**
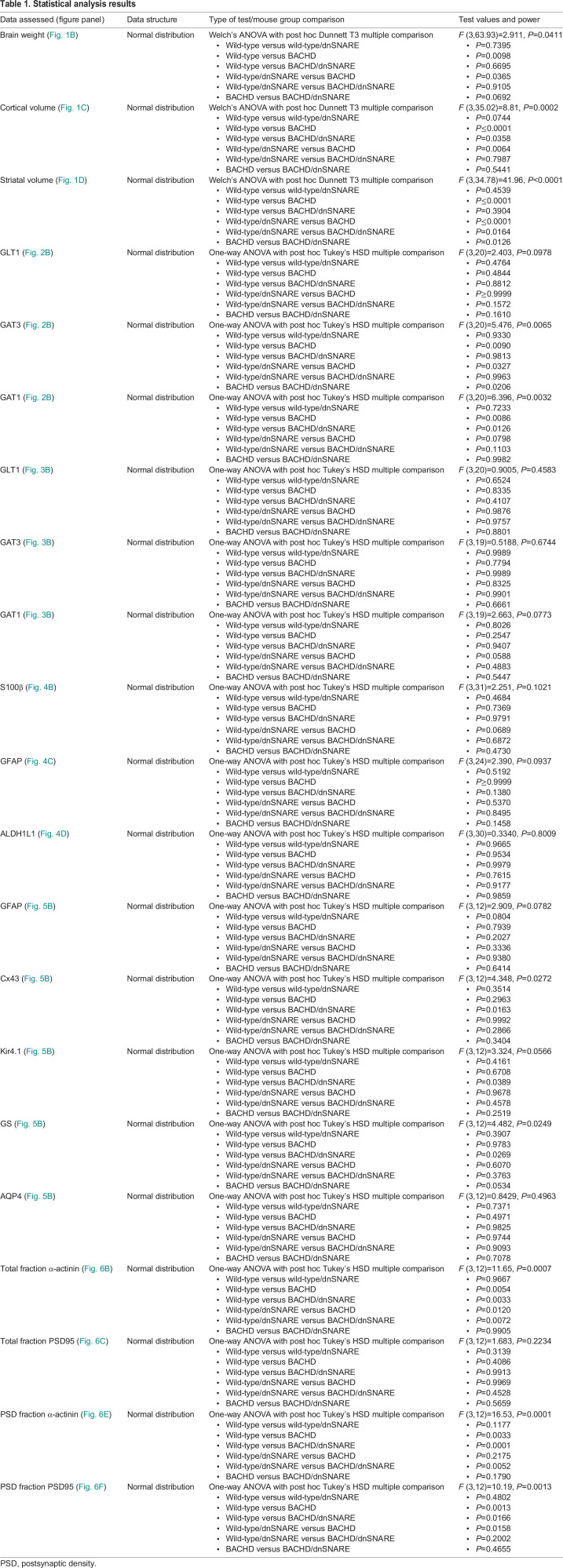
Statistical analysis results

There were significant differences between the genotypes [*F* (3,34.78)=41.96, *P*<0.0001] for striatal volume (Fig. 1D). BACHD mice had reduced striatal volume compared to that of wild-type and wild-type/dnSNARE mice (*P*<0.0001). The BACHD/dnSNARE mice also had reduced striatal volume compared to that of wild-type/dnSNARE mice (*P*=0.0164). The striatal volume of BACHD/dnSNARE mice was significantly increased compared to that of BACHD mice (*P*=0.0126). Thus, reducing SNARE-dependent exocytosis in BACHD mice protected against striatal volume decrease when comparing BACHD mice to BACHD/dnSNARE mice (*P*=0.0126).

### Amino acid transporter expression is altered in striatal subcellular fractions

**Fig. 2.**
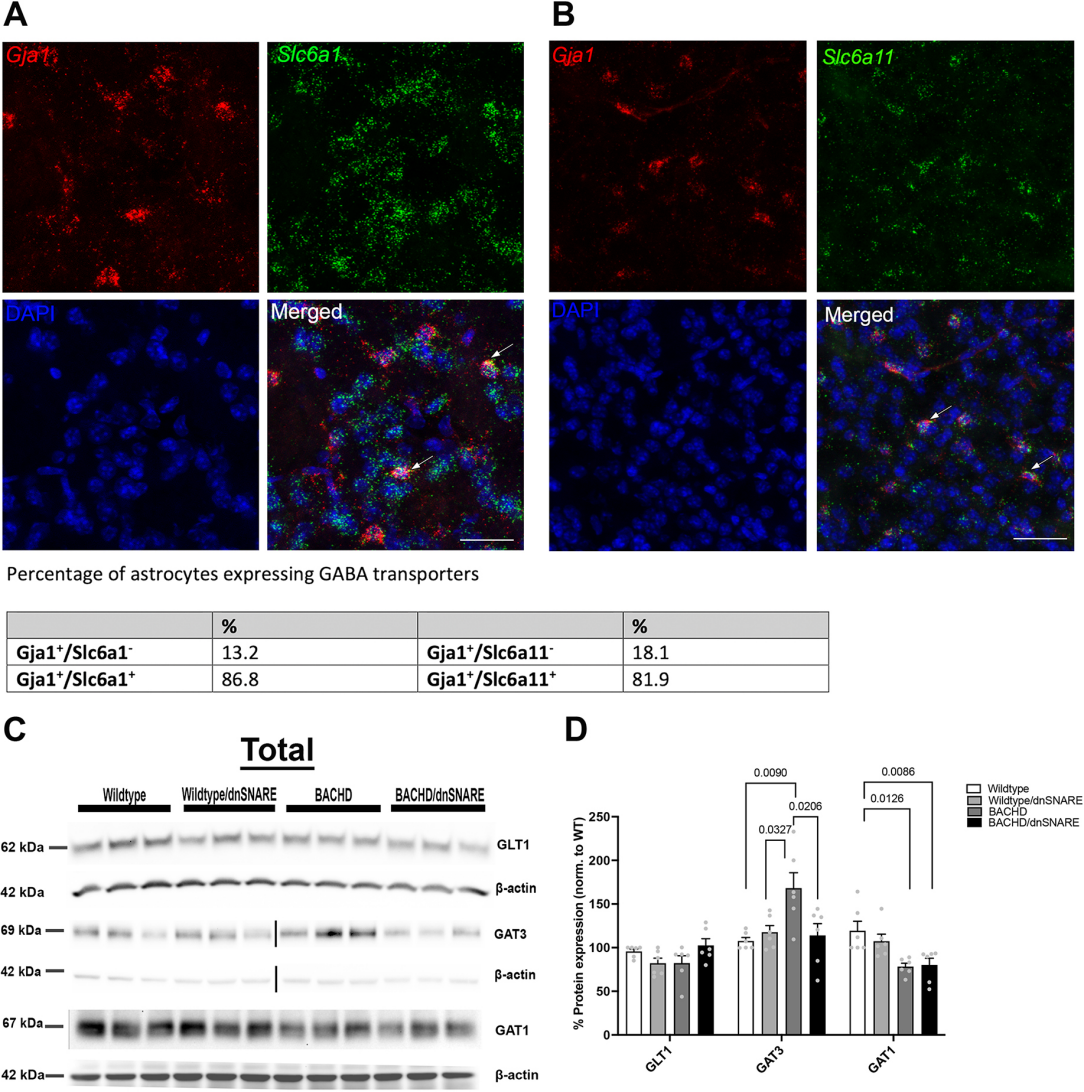
**Expression of neurotransmitter transporters.** (A,B) Representative RNAscope images of *Slc6a1*, *Slc6a11*and *Gja1* in the striatum. Scale bars: 50 μm. The numbers of *Gja1*-positive cells co-labeled with *Slc6a1* (encoding GAT1) or *Slc6a11* (encoding GAT3) were counted. (C) Representative western blots of GLT1, GAT3 and GAT1 protein expression in the striatum of wild-type, wild-type/dnSNARE, BACHD and BACHD/dnSNARE mice (each lane represents a single sample; each sample is pooled from three mice of the same genotype). (D) Western blot quantification of GLT1, GAT3 and GAT1 (*n*=6/genotype). WT, wild type. The loading control β-actin shown for GAT1 in C is the same as the β-actin shown for Cx43 in Fig. 5A. The same membrane was probed for β-actin, GAT1 and Cx43. The loading control β-actin shown for GLT1 in C is the same as the β-actin used for GFAP in Fig. 5A. GLT1 and GFAP were probed on the same blot membrane. Data are mean±s.e.m. Differences among the groups were assessed by one-way ANOVA followed by Tukey's HSD multiple comparison procedure. Non-significant *P*-values are not displayed on graphs. Refer to Table 1 for all *P*-values.

There is accumulating evidence that the GABAergic system is altered in mouse models of HD and HD patients (Spokes et al., 1980; Cepeda et al., 2003; André et al., 2011; Garret et al., 2018; Hsu et al., 2018). Astrocytes can detect transmitters released from neurons and can modulate synaptic transmission by releasing gliotransmitters (Araque et al., 2014; Verkhratsky et al., 2016). Astrocyte and neuronal transporters contribute to the homeostasis of the cellular environment by taking up transmitters from the synaptic cleft. The GABA transporter GAT3 is primarily expressed in astrocytes (Minelli et al., 1996). Neurons and astrocytes express GAT1 (Minelli et al., 1995; Jin et al., 2011). To validate previous cellular localization of *Slc6a1* (encoding GAT1) or *Slc6a11* (encoding GAT3) mRNAs, we used RNAscope probes targeted to *Gja1* to label astrocytes along with probes targeted to *Slc6a1* or *Slc6a11*. We counted the number of *Gja1*-positive cells co-labeled with either *Slc6a1* (Fig. 2A) or *Slc6a11* (Fig. 2B). We observed that ∼87% of *Gja1*-positive cells were *Slc6a* positive in the striatum. Co-labeling of *Gja1* and *Slc6a11* revealed that ∼81% of cells positive for *Gja1* were also positive for *Slc6a11* in the striatum. We determined the protein expression of GABA transporters in BACHD mice and assessed whether reducing astrocyte SNARE-dependent exocytosis would influence GABA transporter expression (Fig. 2C,D).

**Fig. 3.**
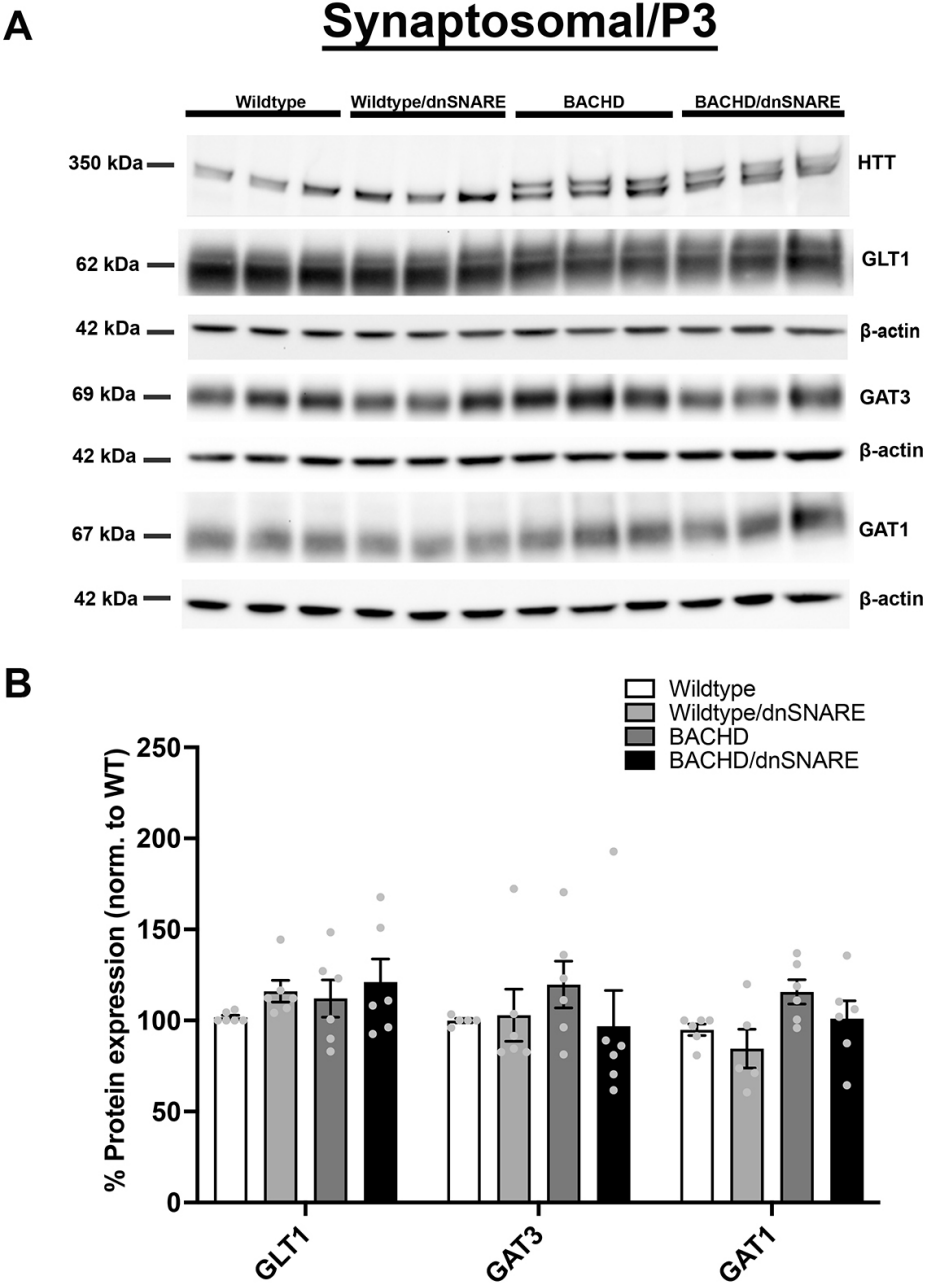
**Glutamate and GABA transporter levels in the synaptosomal fraction.** (A) Representative western blots of glutamate and GABA transporter protein expression in wild-type, wild-type/dnSNARE, BACHD and BACHD/dnSNARE mice. (B) Western blot quantification of the transporters (shown are *n*=3 independent samples). HTT was probed to confirm the genotype of sample mice on the same blot probed for GAT1 (same β-actin loading control as shown for GAT1). No significant difference was observed in GLT1, GAT3 and GAT1 (*n*=5-6/genotype). Data are mean±s.e.m. Differences among the groups were assessed by one-way ANOVA followed by Tukey's HSD multiple comparison procedure. Non-significant *P*-values are not displayed on graphs. Refer to Table 1 for all *P*-values.

Previous RNA analyses showed that excitatory amino acid transporter 2 (*EAAT2*; *SLC1A2*) was reduced in HD patient striatal tissue (Arzberger et al., 1997; Estrada-Sánchez and Rebec, 2012). A reduction in the protein expression of GLT1/EAAT2 was observed in mouse models of HD (Estrada-Sánchez et al., 2009; Petr et al., 2013). Although data from patient tissue and various mouse models have demonstrated a decrease in the glutamate transporter GLT1, we did not observe this decrease by western blotting using a total homogenate extracted from the striatum of BACHD mice (Fig. 2D). We did, however, observe significant differences in GAT3 [*F* (3,20)=5.476, *P*=0.0065] protein expression among the genotypes. GAT3 expression levels were significantly increased in BACHD mice compared to wild-type (*P*=0.0090) and wild-type/dnSNARE (*P*=0.0327) mice. Interestingly, the level of GAT3 in BACHD/dnSNARE mice was significantly different from that in BACHD mice (*P*=0.0206), like the levels observed in wild-type mice (Fig. 2D). Our analysis revealed a significant difference in GAT1 [*F* (3,20)=6.396, *P*=0.0032] protein expression among the genotypes. The expression of GAT1 in BACHD (*P*=0.0086) and BACHD/dnSNARE (*P*=0.0126) mice was reduced compared to that in wild-type mice (Fig. 2D). Given that transporters are prominently localized to synaptic regions and that the compartmental expression could be differentially affected, we assessed transporter expression in the synaptosomal (P3) fraction (contains synaptic proteins, synaptic membranes and astrocytic membranes). We did not observe statistically significant differences in GLT1, GAT3 or GAT1 protein levels in the synaptosomal fraction (Fig. 3A,B).

### Number of S100β-, GFAP- and ALDH1L1-positive astrocytes

**Fig. 4.**
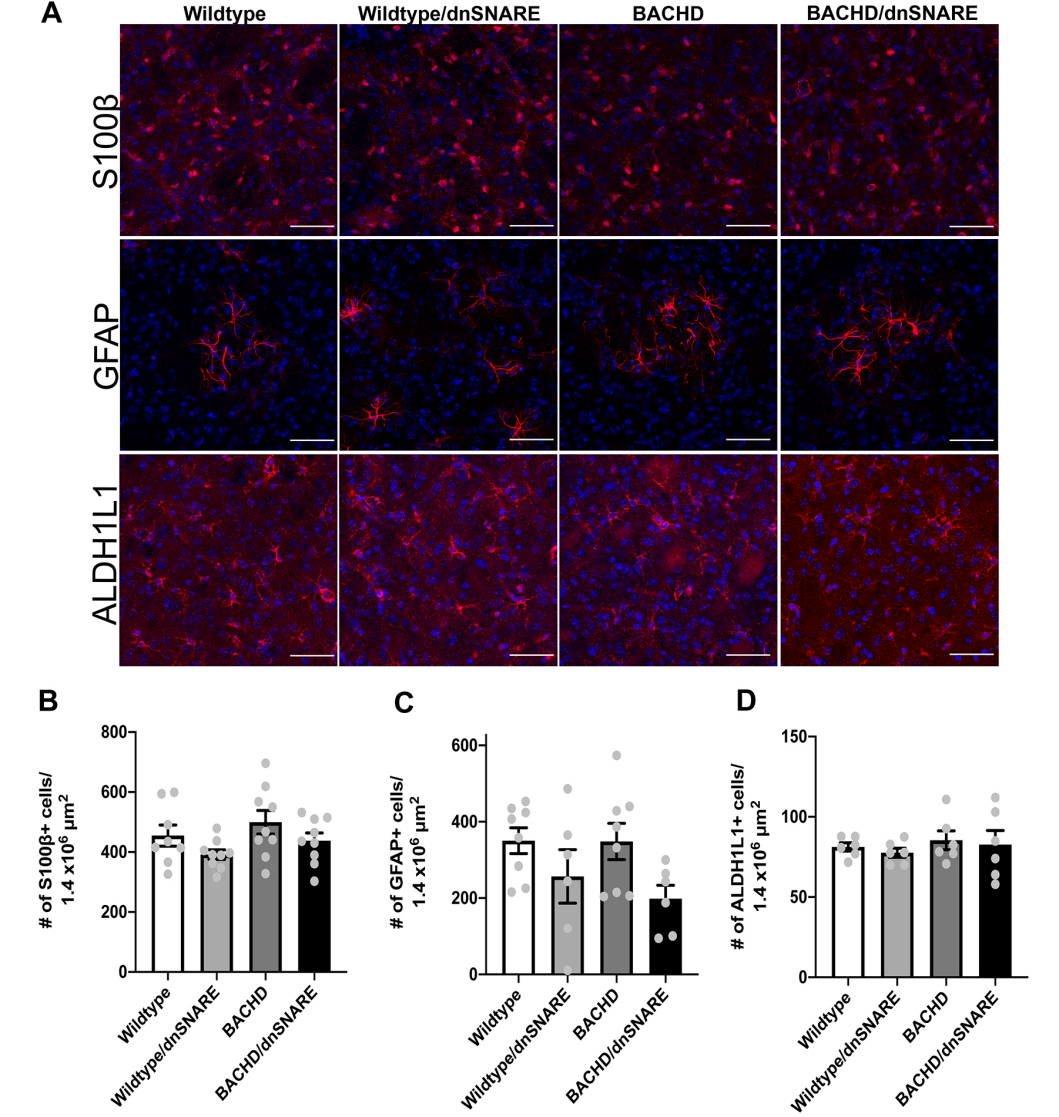
**Number of S100β-, GFAP- and ALDH1L1-positive astrocytes in the striatum in wild-type, wild-type/dnSNARE, BACHD and BACHD/dnSNARE mice.** (A) Immunofluorescence staining of S100β-, GFAP- and ALDH1L1-positive astrocytes (red) in wild-type, wild-type/dnSNARE, BACHD and BACHD/dnSNARE tissues. Scale bars: 50 μm. (B-D) No change in S100β-positive (B), GFAP-positive (C) or ALDH1L1-positive (D) astrocyte numbers was observed in these mice. Blue is DAPI staining (*n*=6-9/genotype). Data are mean±s.e.m. Differences among the groups were assessed by one-way ANOVA followed by Tukey's HSD multiple comparison procedure. Non-significant *P*-values are not displayed on graphs. Refer to Table 1 for all *P*-values.

Astrocyte reactivity is a hallmark of many neurological disorders (Ben Haim et al., 2015; Phatnani and Maniatis, 2015). Analysis of postmortem HD tissues showed significant astrogliosis with increasing disease grade (based on striatal degeneration) as defined by increased GFAP expression and altered astrocyte morphology (Faideau et al., 2010). Most of the mHTT-expressing mouse models do not have overt astrocyte reactivity even when HD phenotypes are apparent (Mangiarini et al., 1996; Tong et al., 2014; Haim et al., 2015; Khakh et al., 2017). To further assess whether the reduction in astrocyte SNARE-dependent exocytosis affects any other neuropathological features, we analyzed mice at 15 months of age to assess astrocyte number. We used immunohistochemistry to stain striatal tissues for astrocytes using antibodies against S100β, GFAP and ALDH1L1 (astrocytic markers). We did not observe any significant differences in S100β, GFAP or ALDH1L1 staining intensity (Fig. 4A) or in positive astrocyte numbers among the genotypes (Fig. 4B-D). Thus, mHTT in BACHD mice does not cause increased astrocyte reactivity, as indicated by a change in GFAP expression, or affect astrocyte number. Furthermore, there is no change in astrocyte number after reducing SNARE-dependent exocytosis in wild-type or BACHD mice.

### Expression of astrocyte-enriched proteins

**Fig. 5.**
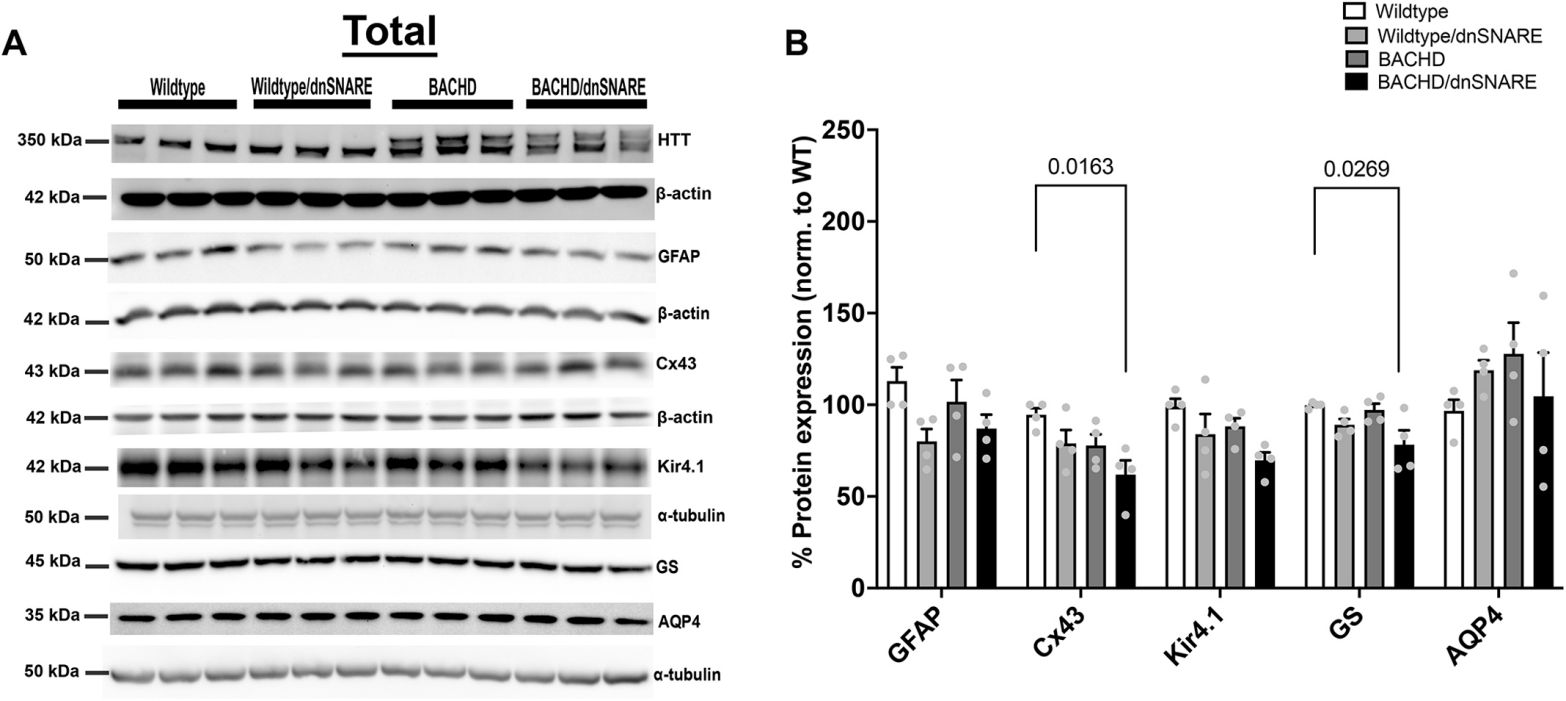
**Astrocyte-enriched protein expression in the striatal total cell homogenate.** (A) Representative western blots of various astrocyte-enriched proteins in the striatal total cell homogenate of wild-type, wild-type/dnSNARE, BACHD and BACHD/dnSNARE mice. (B) Quantification of the western blot results (*n*=4/genotype). HTT was probed to confirm the genotype of our mouse models. The loading control β-actin shown for Cx43 in A is the same as the β-actin shown for GAT1 in Fig. 2C. The same membrane was probed for β-actin, GAT1 and Cx43. The loading control β-actin shown for GFAP in A is the same as the β-actin used for GLT1 in Fig. 2C. The same membrane was probed for β-actin, GLT1 and GFAP. Data are mean±s.e.m. Differences among the groups were assessed by one-way ANOVA followed by Tukey's HSD multiple comparison procedure. Non-significant *P*-values are not displayed on graphs. Refer to Table 1 for all *P*-values.

In HD, there are many molecular and functional changes that occur in astrocytes that can contribute to disturbance in the cellular environment (Cahoy et al., 2008; Tong et al., 2014; Verkhratsky et al., 2014; Phatnani and Maniatis, 2015; Khakh et al., 2017; Li et al., 2019; Zhou et al., 2019). To assess the levels of astrocyte-enriched proteins in BACHD/dnSNARE, BACHD, wild-type/dnSNARE and wild-type mice, we performed western blot analysis of total homogenate of the striatum for GFAP, connexin 43 (Cx43; encoded by *Gja1*), ATP-dependent inwardly rectifying potassium channel (Kir4.1; encoded by *Kcnj10*), glutamine synthetase (GS; encoded by *Glul*), and aquaporin4 (AQP4) (Fig. 5A,B). We did not observe any differences in GFAP, Kir4.1 or AQP4 protein expression among the genotypes. We observed a significant difference in Cx43 protein expression in BACHD/dnSNARE mice compared to wild-type mice (*P*=0.0163). We also observed a significant difference in GS protein expression in BACHD/dnSNARE mice compared to wild-type mice (*P*=0.0269). Thus, reducing astrocytic SNARE-dependent exocytosis does not cause changes in astrocyte-enriched protein expression in wild-type or BACHD mice.

**Fig. 6.**
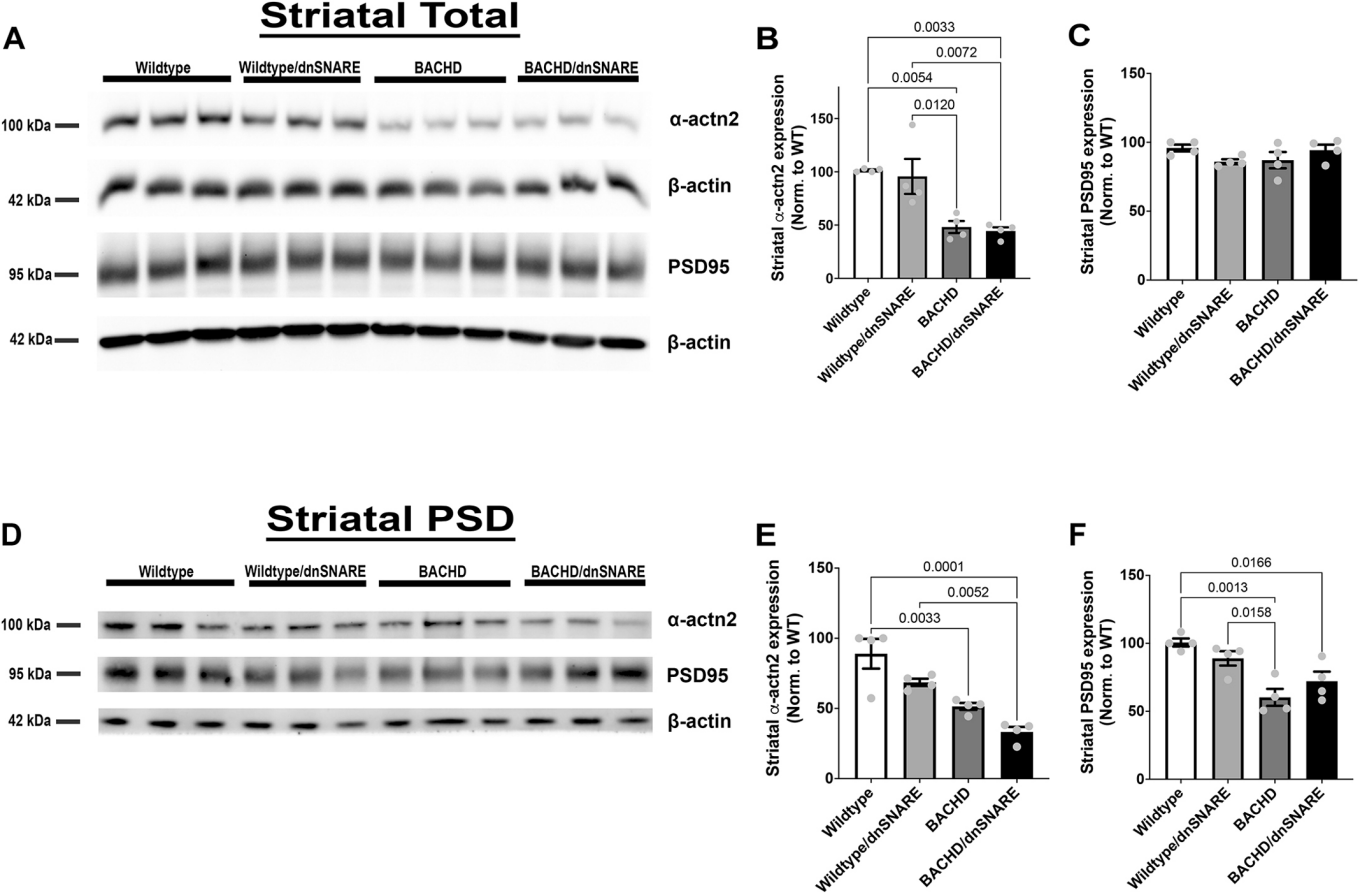
**Reduction in SNARE-dependent exocytosis in BACHD mice had no effect on striatal ACTN2 or PSD95 expression.** (A) Representative western blots of total homogenate for ACTN2 and PSD95 (*n*=4/genotype). (B,C) Quantitation of the western blots for ACTN2 (B) and PSD95 (C) in the total homogenate fraction (*n*=4/genotype). (D) Representative blots of ACTN2 and PSD95 in the postsynaptic density (PSD) fractions (*n*=4/genotype). (E,F) Quantitation of the western blots for ACTN2 (E) and PSD95 (F) in the PSD fractions (*n*=4/genotype). Data are mean±s.e.m. Differences among the groups were assessed by one-way ANOVA followed by Tukey's HSD multiple comparison procedure. Non-significant *P*-values are not displayed on graphs. Refer to Table 1 for all *P*-values.

The HTT protein can influence synaptic protein localization including that of PSD95 (DLG4) (Parsons et al., 2014). In a previous study, silencing HTT in fibroblasts blocked the recruitment of α-actinin 2 (ACTN2) to the membrane, suggesting that HTT interacts with ACTN2 and is involved in cytoskeleton/actin functions (Tousley et al., 2019). Synaptic protein expression is decreased in HD patients and mouse models of HD (DiProspero et al., 2004; Wang et al., 2014; Wood et al., 2019; Sapp et al., 2020). When mHTT in astrocytes was reduced using a GFAP-CreER^T2^ mouse model, ACTN2 and PSD95 expression were increased compared to that in BACHD mice, thus implicating astrocytes in HD pathogenesis (Wood et al., 2019). In this study, we examined whether changes in SNARE-dependent exocytosis would contribute to changes in ACTN2 and PSD95 expression, as previously observed when mHTT expression was reduced in astrocytes. We used western blot analyses to assess PSD95 and ACTN2 expression in the striatal total homogenate and postsynaptic density (PSD) fractions from the BACHD/dnSNARE, BACHD, wild-type/dnSNARE and wild-type mice. We observed a significant difference in ACTN2 expression among the genotypes in the total fraction [*F* (3,12)=11.65, *P*=0.0007] (Fig. 6A,B). Both BACHD and BACHD/dnSNARE mice had reduced ACTN2 expression in the total fraction compared to that in wild-type (*P*=0.0054 and *P*=0.0033, respectively) or wild-type/dnSNARE (*P*=0.0120 and *P*=0.0072, respectively) mice. In BACHD/dnSNARE mice, ACTN2 expression was not significantly different from that in BACHD mice. We observed no significant differences in PSD95 protein expression in the total homogenate among the genotypes (Fig. 6C).

Because ACTN2 and PSD95 are enriched in the PSD (Fig. 6D), we analyzed the PSD fraction using western blotting. We observed a significant difference in ACTN2 protein expression among the genotypes in the PSD fraction [*F* (3,12)=16.53, *P*=0.0001] (Fig. 6E). Both BACHD mice and BACHD/dnSNARE mice had reduced ACTN2 expression compared to wild-type mice (*P*=0.0033 and *P*=0.0001, respectively). BACHD/dnSNARE mice also had reduced ACTN2 protein expression compared to that in wild-type/dnSNARE mice (*P*=0.0052), but there was no significant difference in ACTN2 expression between BACHD/dnSNARE and BACHD mice. We observed a significant difference in PSD95 protein expression in the PSD fraction among the genotypes [*F* (3,12)=10.19, *P*=0.0013] (Fig. 6F). BACHD mice had reduced PSD95 protein expression compared to that in wild-type (*P*=0.0013) and wild-type/dnSNARE (*P*=0.0158) mice. BACHD/dnSNARE mice had reduced PSD95 protein expression compared to that in wild-type (*P*=0.0166) mice. There was no significant difference in PSD95 protein expression between BACHD/dnSNARE and BACHD mice. Thus, reducing SNARE-dependent exocytosis does not cause changes in ACTN2 or PSD95 expression.

## DISCUSSION

This study provides insight into the contribution of SNARE-dependent exocytosis from mHTT-expressing astrocytes to neuropathological changes in HD. We used dnSNARE mice to reduce astrocyte SNARE-dependent exocytosis in the brains of BACHD mice and assessed neuropathological changes at 13-15 months of age. We observed a statistically significant increase in the striatal volume of the BACHD/dnSNARE mice compared to that in BACHD mice at 13-15 months of age. We did not observe statistically significant changes in brain weight or cortical volume in the BACHD/dnSNARE mice, although both were increased. When mHTT was reduced in astrocytes of BACHD/GFAP-CreER^T2^ mice, there was significant improvement in brain weight and striatal volume compared to that in BACHD mice, although changes in cortical volume did not reach statistical significance (Wood et al., 2019). Striatal volume is significantly decreased in HD; thus, our observation of increased striatal volume in BACHD/dnSNARE mice suggests that reducing astrocytic SNARE-dependent exocytosis is protective against striatal atrophy.

Astrocytes contribute to the clearance of amino acids such as glutamate and GABA by expressing transporters such as GLT1, GAT1 and GAT3. This work reveals an increase in GAT3, and a decrease in GAT1, levels in total homogenate in the striatum of BACHD mice and no changes in GLT1, GAT3 or GAT1 levels in the synaptosomal fraction. The striatal GAT1 total homogenate levels in the BACHD/dnSNARE mice were the same as those in the BACHD mice. In BACHD/dnSNARE mice, the total homogenate level of GAT3 was reduced compared to that in BACHD mice and not significantly different from that in wild-type mice. The level of GAT1 in the total striatal homogenate from BACHD mice is not altered by the expression of dnSNARE; previous studies using the dnSNARE model found no changes in the total or surface expression of GLT1 (Fellin et al., 2009). Importantly, we did not observe changes in the levels of GLT1, GAT1 or GAT3 in the total or membrane (synaptosomal/P3) fraction in wild-type/dnSNARE mice compared to wild-type mice. It is possible that the increase in GAT3 is a compensatory change in BACHD mice in response to the decrease in GAT1 to regulate the levels of extracellular GABA, as previous studies revealed a role for GAT3 in modulation of GABAergic neurotransmission when GAT1 was blocked (Wójtowicz et al., 2013). However, the modulation of GAT3 levels in the presence of dnSNARE in BACHD mice decreases the likelihood of this being merely a compensatory response. Our modulation of SNARE-dependent exocytosis is specific to astrocytes, and thus there would likely be more alterations observed in astrocyte-enriched proteins like GAT3. In this study, we did not differentiate between GAT1 levels in astrocytes and neurons owing to the nature of our subcellular fractionation. However, GAT1 levels are significantly higher in neurons than in astrocytes (Zhou and Danbolt, 2013). Although we observed significantly less GAT3 in total homogenate extracted from BACHD/dnSNARE mice than in total homogenate extracted from BACHD mice, no statistically significant change in the synaptosomal/P3 fraction was observed. Given the localization of GAT3 throughout the cellular membrane in astrocytes and not only in the region directly adjacent to synapses, it is also likely that this change would be most prominently observed in the total protein homogenate.

A previous study showed no change in GLT1 or Kir4.1 in BACHD or BACHD/GFAP-CreER^T2^ mice (Wood et al., 2019). In agreement with that study, we did not observe changes in astrocyte-enriched proteins GLT1, GFAP, Kir4.1, AQP4 (M1 isoform) or GS in the BACHD mice. We recognize that there is a reduction in GLT1/EAAT2 and Kir4.1 expression in the striatum in other mHTT-expressing mouse models (Liévens et al., 2001; Tong et al., 2014; Khakh et al., 2017), and increased GFAP expression in the HD lentiviral mouse model (Faideau et al., 2010; Wu et al., 2020). Other studies found a decrease in the mRNA and protein expression levels of GS in an HD mouse model (Liévens et al., 2001; Tong et al., 2014). Most of those changes were observed in the rapidly progressing R6/2 model, which contains the N-terminal fragment of mHTT, whereas the BACHD mouse model is a very slowly progressing model and these changes may not be readily apparent in this full-length model at this age. Furthermore, the analyses performed here are on extracted striatal protein, and thus subtle differences that may be seen at a cellular level using other methodologies may not be detected.

Astrocytic SNARE-dependent exocytosis is involved in various processes, such as synaptic transmission, plasticity, neuronal excitability and synaptogenesis (Araque et al., 1999; Angulo et al., 2004; Araque et al., 2014; Khakh and Sofroniew, 2015). Astrocytes in diseased states lose their protective/supportive roles, thus further disturbing the neuronal environment through altered calcium (Ca^2+^) homeostasis and release of molecules (Phatnani and Maniatis, 2015; Li et al., 2019). One study showed that the release of ATP from astrocytes and neurons regulates NMDA receptors through PSD95 multi-protein complex (Lalo et al., 2016). There are changes in synaptic protein expression in HD patient tissue (DiProspero et al., 2004; Fourie et al., 2014). Both PSD95 and ACTN2 are critical components of the PSD and important for proper neurotransmission. ACTN2 has been shown to anchor PSD95 at postsynaptic sites and is critical for postsynaptic organization (Hodges et al., 2014; Matt et al., 2018). HTT interacts directly with PSD95, and it is known that PSD95 and ACTN2 are decreased in mouse models including BACHD (Cha, 2007; Wang et al., 2014; Wood et al., 2019; Sapp et al., 2020). When mHTT was reduced in astrocytes in BACHD/GFAP-CreER^T2^ mice, expression of PSD95 and ACTN2 was restored to normal levels (Wood et al., 2019). That study also showed that the NMDA receptor-mediated current was normalized in the BACHD/GFAP-CreER^T2^ mice (Wood et al., 2019). Taken together, the findings show that reducing mHTT in astrocytes normalizes synaptic abnormalities. When we reduced SNARE-dependent exocytosis in astrocytes using BACHD/dnSNARE mice, there were no overt changes in the PSD proteins PSD95 or ACTN2. In this case, reducing SNARE-dependent exocytosis in astrocytes did not contribute to changes in synaptic protein expression, thus suggesting that neuroprotection in BACHD mice may occur through an astrocytic mechanism other than SNARE-dependent exocytosis.

When astrocytic SNARE-dependent exocytosis was reduced in BACHD mice, improvement in motor coordination and worsening of the anxiety-like phenotype was found, but no effect on the depressive-like phenotype was observed (King et al., 2020). Thus, we recognize that broadly targeting astrocyte SNARE-dependent exocytosis would not be the most viable approach to modulate these behaviors. Nonetheless, in the present study, we showed that there is a significant increase in striatal volume, and a non-statistically significant increase in brain weight and cortical volume, in the BACHD/dnSNARE mice. Although our study does not reveal which transmitters or molecules released by astrocytes contribute to the striatal atrophy observed in BACHD mice, it does suggest that reducing SNARE-dependent exocytosis is protective against striatal atrophy observed in the BACHD mouse model.

## MATERIALS AND METHODS

### Animals

All animal procedures were performed in accordance with the National Institutes of Health Guide for the Care and Use of Laboratory Animals and were approved by the University of Alabama at Birmingham (UAB) Institutional Care and Use Committee.

BACHD/wild-type mice were maintained by breeding with FvB/NJ mice (The Jackson Laboratory). Dr Philip Haydon and Dr Vladimir Parpura provided hGFAP: tTA, *tetO*: dnSNARE/*tetO*: eGFP mouse strains. The GFAP.tTa and tetO.SNARE mice were maintained on the C57BL6 background (The Jackson Laboratory). The dnSNARE mouse model was generated by crossing GFAP.tTa and tetO.SNARE mouse lines. The GFAP.tTA/tetO.SNARE (dnSNARE) (Pascual et al., 2005) mice were bred to the BACHD mice to generate F1 BACHD/dnSNARE mice used in these studies.

### Doxycycline paradigm

The dnSNARE and BACHD/dnSNARE breeding mice and their progeny after weaning, along with appropriate mice (wild-type and BACHD) for comparison, were maintained on chow containing doxycycline (625 mg/kg; Envigo Teklad) to suppress transgene expression until 7.5 months of age. The mice were removed from doxycycline-containing food pellets and fed standard mouse chow to allow transgene expression at 7.5 months of age.

### Brain weight

Mice were perfused with 4% paraformaldehyde (PFA; Sigma-Aldrich, P6148). The brains of the mice were dissected and postfixed overnight at 4°C in 4% PFA. Brains were placed in 30% sucrose until they sank at 4°C. Excess sucrose was removed from the brain to prevent any weighing errors. The forebrain and cerebellum were weighed.

### Cresyl Violet staining

Wild-type, wild-type/dnSNARE, BACHD and BACHD/dnSNARE were perfused with 4% PFA. The brains were sectioned at 40 μm using a Leica SM 2010K microtome. The first section was chosen at random from the first ten sections containing striatum, and every tenth section thereafter was dried on microscope slides (Fisher Scientific, 12-550-15) for 2-3 days. Tissues were dehydrated in 70%, 95%, 100% ethanol for 3 min each, and this step was repeated. Tissues were then placed in xylene (clearing agent) for 10 min, and rehydrated in 100% ethanol (2×), 95% ethanol (1×) and 70% ethanol (1×) for 3 min each. They were then placed in distilled water for 1 min and in Cresyl Violet (Electron Microscopy Sciences, 12780) solution for three dips (5 s each), and de-stained by dunking in tap water five times with movement. The tissues were then placed in 70% (1×), 95% (1×) and 100% (1×) ethanol for 3 min each, placed in xylene for 10 min, coverslipped with Permount (Fisher Scientific, SP15-500) and allowed to dry overnight at room temperature.

### Stereology and brain volume estimation

Wild-type, wild-type/dnSNARE, BACHD and BACHD/dnSNARE mice were perfused with 4% PFA (Sigma-Aldrich, P6148), and 40 μm coronal brain sections were cut using a Leica SM 2010K microtome. The first section was chosen at random from the first ten sections containing striatum, and every tenth section thereafter was dried on microscope slides (Fisher Scientific, 12-550-15) for 2 days and then stained with Cresyl Violet (Electron Microscopy Sciences, 12780) to highlight neuronal structures. Sections were mounted and coverslipped with Permount (Fisher Scientific, SP15-500). Stereo Investigator software (Microbrightfield, MBF Bioscience, Williston, VT, USA) and the Cavalieri method were used to determine the total volume of the striatum and cortex at 13-15 months of age.

### Tissue processing and confocal imaging

Mice were perfused with 4% PFA (Sigma-Aldrich, P6148). The brains of the mice were dissected and postfixed overnight at 4°C in 4% PFA, followed by cryoprotection in 30% sucrose at 4°C. Brains were sectioned with a microtome and stored in cryopreserve (0.1 M PBS, 0.01 M MgCl_2_ and 0.5 M sucrose, 50% glycerol).

### RNAscope

Fluorescence *in situ* hybridization was performed using an RNAscope® Fluorescent Multiplex Detection v1 kit. Brains were flash frozen to prepare 20 µm coronal sections using a cryostat (Leica CM 1950). Sections were placed onto SuperFrost Plus microscope slides (Fisher Scientific, 12-550-15), which were immediately placed on dry ice and stored at −80°C. Slides were incubated in a glass slide holder in 4% PFA in 1× PBS (pH 7.4) at 4°C for 15 min directly from the −80°C freezer. Sections were dehydrated in a stepwise fashion using 50%, 70% and 100% ethanol solutions diluted in diethyl pyrocarbonate (DEPC; Sigma-Aldrich, D5758)-treated ddH_2_O. A hydrophobic barrier pen (Vector Laboratories, H-4000) was used to draw around each section, and proteins were digested using protease IV (Advanced Cell Diagnostics, 322,336) for 5 min. Slides were subsequently immersed in a 1× PBS solution for 10 min. Probes targeting the genes for mutant human *HTT* (Advanced Cell Diagnostics, 473,201), *Gja1* (Advanced Cell Diagnostics, 486191-C2), *Slc6a1* (Advanced Cell Diagnostics, 444071-C3) and *Slc6a11* (Advanced Cell Diagnostics, 492661-C3) were used. Slides were incubated in a humidified slide box and washed between each amplification step using a 1× wash buffer (Advanced Cell Diagnostics, 320058). Slides were mounted using ProLong^TM^ Diamond Antifade Mountant with 4′,6-diamidino-2-phenylindole (DAPI; Invitrogen, P36971). Solutions were diluted using DEPC-treated ddH_2_O. The wash buffer was diluted using nuclease-free water (Invitrogen, 10977-023). Cells were counted using ImageJ.

### S100β, GFAP and ALDH1L1 staining

Sections were rinsed 3×10 min in 0.01 M PBS, blocked with 2% normal serum/3% bovine serum albumin in 0.01 M PBS and incubated with antibodies against GFAP (1:500; rabbit, Agilent Technologies, Z033429-2; RRID:AB_10013382) or S100β (1:500; rabbit, Abcam, ab52642; RRID:AB_882426) or ALDH1L1 (1:500; rabbit, Abcam, ab87117; RRID:AB_10712968) overnight at 4°C. Tissue was incubated for 2 h with appropriate secondary antibodies, donkey anti-goat Alexa Fluor 488 (1:500; Invitrogen, A11055) or goat anti-rabbit Alexa Fluor 555 (1:500; Invitrogen, A21428). Images of bilateral striatum were taken using a Nikon Eclipse Ti epi-fluorescent microscope through a Nikon Plan Fluor 10×/0.30 NA (∞/0.17 WD 16) objective. Acquired images of bilateral striatum were counted for S100β-, GFAP- and ALDH1L1-positive astrocytes. ImageJ Fiji was used to obtain cellular counts. Images for Fig. 4 were acquired using a Nikon Eclipse Ti2-C2 confocal microscope and parameters in accordance with the Shannon–Nyquist theorem. Images were acquired through a Nikon Plan Apo λ 20×/0.75 NA (OFN25 DIC N2) objective and were electronically zoomed in at 2.61× and processed utilizing NIS-Elements software (Nikon, Tokyo, Japan).

### Subcellular fractionation for western blotting

Subcellular fractionation was performed as described in Au-Bermejo et al. (2014) with slight modification (Fig. S1). Adult mouse striatum was collected at 12 months. Striatal tissue from three mice was pooled for each sample to obtain sufficient protein for use in western blot analyses after subcellular fractionation. Tissues were homogenized in 4 ml sucrose buffer (0.32 M sucrose, 4 mM HEPES and Pierce protease inhibitor). Total homogenate (T; 100 μl collected) was centrifuged in a fixed angle rotor at 900 ***g*** for 10 min at 4°C, and the pellet (P1) was discarded. The supernatant (S1; 100 μl collected) was transferred to new 50 ml conical tube. Remaining S1 was centrifuged at 10,000 ***g*** for 15 min at 4°C (Beckman Coulter Avanti® J-E Centrifuge, JA-12 rotor). Subsequently,1 ml of the supernatant (S2) was collected, and the remainder was discarded. The resulting pellet (P2) was solubilized in 1 ml sucrose buffer and an additional 3 ml sucrose buffer was added. The solubilized P2 was then centrifuged a second time with the same parameters. The supernatant (S′) was discarded. The resulting pellet (P2′) was solubilized in 4 ml ddH_2_O and transferred to a 15 ml tapered glass-Teflon homogenizer, and 200 μl of the P2′ was reserved. Samples were homogenized by hand and then transferred to 50 ml conical tubes with 16 μl 1 M HEPES. The samples were rotated on a shaker for 30 min at 4°C and then centrifuged at 23,200 ***g*** for 20 min at 4°C. One milliliter of the reserved supernatant (S3) was collected and the remainder was discarded. Pellet (P3) was resuspended in 1 ml sucrose buffer and 100 μl was reserved. Remaining P3 sample was layered on top of a discontinuous sucrose gradient (0.8 M, 1.0 M and 1.2 M sucrose, 4 mM HEPES pH 7.4). Samples were ultracentrifuged at 150,000 ***g*** for 2 h at 4°C (BeckMan Optimal™ L-70 K ultracentrifuge, SW 41 Ti rotor). Using an 18G needle and 1 ml syringe, fluid at the 1.0 M/1.2 M interphase was withdrawn. Samples were diluted in 2.5 volumes of 4 mM HEPES and ultracentrifuged at 200,000 ***g*** for 30 min at 4°C (BeckMan Coulter Optima™ Max-XP ultracentrifuge). Pellet was re-suspended in 400 μl HEPES buffer (50 mM HEPES, 2 mM EDTA, Pierce protease inhibitor) (SPM) and stored overnight at −80°C. Samples were thawed on ice, diluted in 2.7 ml triton buffer (0.54% Triton X-100, 50 mM HEPES, 2 mM EDTA) and rotated for 15 min at 4°C. Samples were then centrifuged at 30,100 ***g*** for 20 min at 4°C (Eppendorf Centrifuge 5430 R). One milliliter of the supernatant (TSF) was reserved and the remainder was discarded. Pellets (PSD) were solubilized in 100-200 μl HEPES buffer and 2-5 μl 0.5% SDS.

### Western blotting

Each sample was diluted in 1.25 μl 4× LDS sample buffer (Invitrogen, NP0008) and heated at 70°C for 10 min. Then, 10 μg total protein was loaded per lane in NuPAGE 3-8% Tris-Acetate gel (Thermo Fisher Scientific, EA0378BOX) or Bolt™ 4-12% Bis-Tris Plus gel (Thermo Fisher Scientific, NW04120BOX). Samples were ran then transferred to Immuno-Blot PVDF membrane (Bio-Rad, 1620177). The membrane was blocked for 1 h in 5% non-fat milk-PBST (0.1% Tween 20) and incubated overnight at 4°C with the appropriate primary antibodies: anti-ACTN2 (1:3000; mouse, Sigma-Aldrich, A7811; RRID:AB_476766), anti-PSD95 (1:30,000; Antibodies Incorporated, mouse 75–028; RRID:AB_2292909), anti-HTT2166 (1:3000; mouse, Millipore, MAB2166, RRID:AB_2123255), anti-GLT1 (1:10,000; guinea pig, Millipore, AB1783), anti-GFAP (1:10,000; rabbit, Agilent Technologies, Z033429-2; RRID:AB_10013382), anti-Cx43 (1:3000; rabbit, Sigma-Aldrich, C6219; RRID:AB_476857), anti-Kir4.1 (1:1000; rabbit, Alomone Labs, APC-035; RRID:AB_2040120), anti-GS (1:3000; rabbit, Abcam, ab73593; RRID:AB_2247588), anti-AQP4 (1:5000; rabbit, Millipore, ABN411; RRID:AB_2637077), anti-GAT3 (1:1000; rabbit, Novus Biologicals, NBP191920; RRID:AB_11013253), anti-GAT1 (1:1000; rabbit, Abcam, ab185205; RRID:AB_2889907), anti-β-actin (1:3000; mouse, Sigma-Aldrich, A5316; RRID:AB_476743) and anti-α-tubulin (1:3000; mouse, Sigma-Aldrich, T9026; RRID:AB_477593). HTT was probed to confirm that the samples were from the correct mouse models. Each membrane was incubated with the appropriate horseradish peroxidase-conjugated secondary antibody: goat anti-mouse (1:3000; Jackson ImmunoResearch, 115-035-146), goat anti-rabbit (1:3000; Jackson ImmunoResearch, 111-035-144) and donkey anti-guinea pig (1:3000; Jackson ImmunoResearch, 706-035-148). The β-actin used in Fig. 2C with GAT1 and in Fig. 5A for Cx43 is from the same membrane; GAT1 and Cx43 were probed on this membrane. The β-actin shown in Fig. 2C with GLT1 and in Fig. 5A with GFAP is from the same membrane; GLT1 and GFAP were probed on this membrane. The blots were incubated with Pierce enhanced chemiluminescent blotting substrate (Thermo Fisher Scientific, 32106) and imaged on a Bio-Rad ChemiDoc Imaging System. ImageJ software was used to calculate the densities of the bands.

### Statistical analysis

#### Brain weight

After perfusion, total brain weight (in g) was measured, and forebrain weight was recorded after removal of the cerebellum. The data are expressed as mean±s.e.m. Differences among the groups were assessed by Welch's AVOVA test followed by Dunnett T3 multiple comparison procedure.

#### Brain volume estimation

The Cavalieri method was used to determine the total volume of the striatum and cortex at 13-15 months in wild-type, wild-type/dnSNARE, BACHD and BACHD/dnSNARE mice. The data are expressed as mean±s.e.m. Differences among the groups were assessed by Welch's ANOVA test followed by Dunnett T3 multiple comparison procedure.

#### Western blotting

ImageJ software was used to calculate the intensities of each protein band on the western blots. The intensity of the assessed proteins was determined by normalizing to a wild-type sample (protein of interest and β-actin). Each additional sample thereafter was normalized to the initial wild-type sample to obtain the relative intensity for all other wild-type and BACHD samples. The relative intensity of the protein was divided by the relative intensity of β-actin to obtain the adjusted intensity for wild-type and BACHD mouse samples. The adjusted intensity was used to obtain the percentage expression for all proteins. The data are expressed as mean±s.e.m. Differences among the groups were assessed by one-way ANOVA followed by Tukey's HSD multiple comparison procedure.

#### Astrocyte number

The numbers of astrocytes positive for S100β, GFAP and ALDH1L1 were manually counted in ImageJ Fiji and recorded. The data are expressed as mean±s.e.m. Differences among the groups were assessed by the one-way ANOVA test followed by Tukey's HSD multiple comparison procedure. A full summary of statistical analysis results is provided in Table 1.

## Supplementary Material

10.1242/dmm.052002_sup1 Supplementary information
