# Supplementary material for: Modulation of SNARE-dependent exocytosis in astrocytes improves neuropathology in Huntington's disease
Source: Dis Model Mech. 2024 Nov 11;17(11):dmm052002. doi: 10.1242/dmm.052002 (PMC11583919; doi:10.1242/dmm.052002)
Supplement: Supplementary information [file dmm-17-052002-s1.pdf]

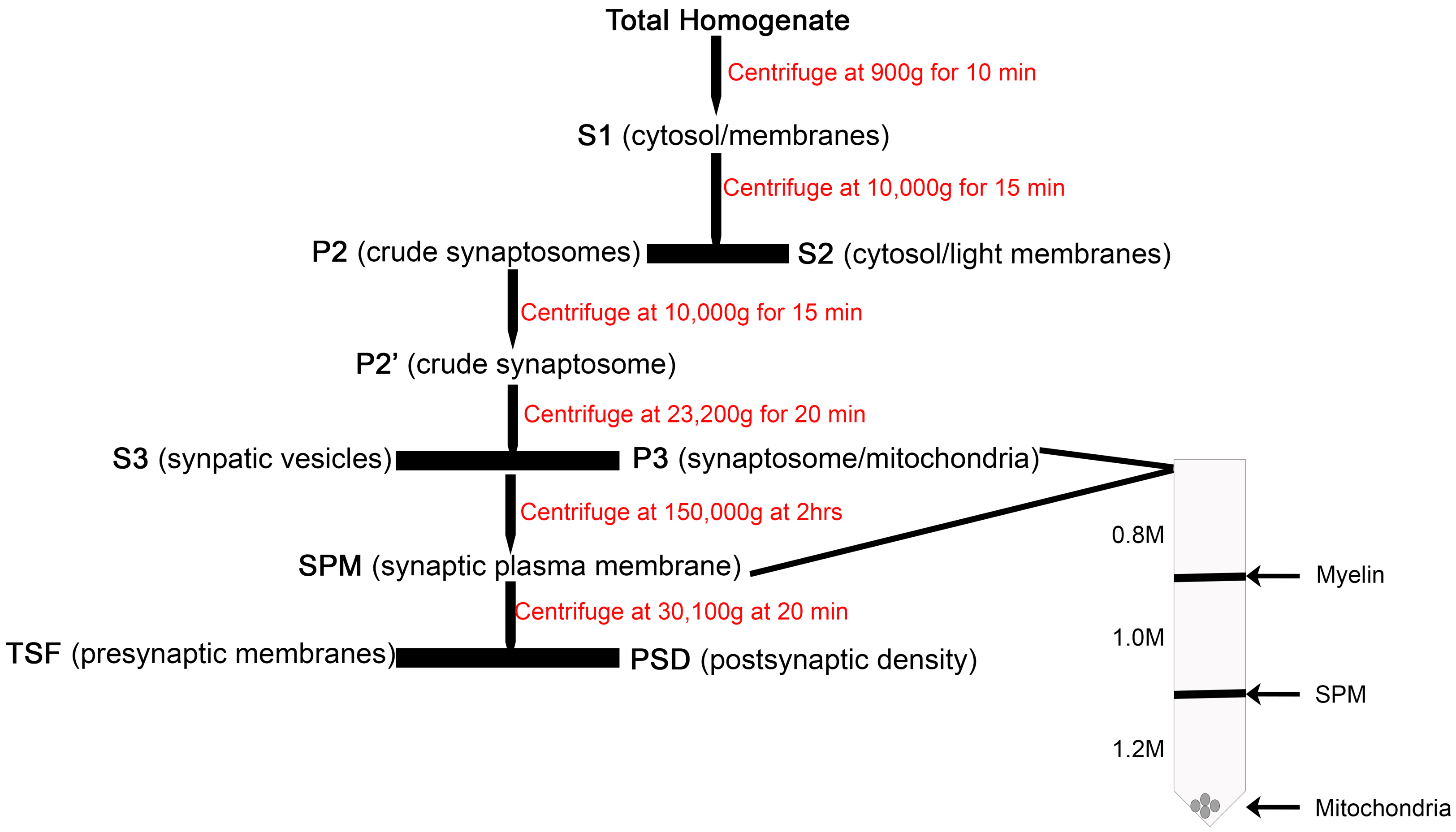
Total Homogenate
Centrifuge at 900g for 10 min
S1 (cytosol/membranes)
Centrifuge at 10,000g for 15 min
P2 (crude synaptosomes)
S2 (cytosol/light membranes)
Centrifuge at 10,000g for 15 min
P2’ (crude synaptosome)
Centrifuge at 23,200g for 20 min
S3 (synpatic vesicles)
P3 (synaptosome/mitochondria)
Centrifuge at 150,000g at 2hrs
SPM (synaptic plasma membrane)
Centrifuge at 30,100g at 20 min
TSF (presynaptic membranes)
PSD (postsynaptic density)
0.8M
1.0M
1.2M
Myelin
SPM
Mitochondria

Fig. S1. Subcellular Fractionation Schematic. Subcellular fractionation is the separation of specific cellular compartments through numerous centrifugations. The fractions are bolded while the centrifugation parameters to obtain each lysate are displayed in red. A diagram is provided to show how P3 centrifugation separates into three fractions: myelin, synaptic plasma membrane (SPM), and mitochondria. SPM is collected to obtain the remaining fractions in this process.

Disease Models & Mechanisms • Supplementary information
